# Laryngeal High-Speed Videoendoscopy: Sensitivity of Objective Parameters towards Recording Frame Rate

**DOI:** 10.1155/2016/4575437

**Published:** 2016-11-21

**Authors:** Anne Schützenberger, Melda Kunduk, Michael Döllinger, Christoph Alexiou, Denis Dubrovskiy, Marion Semmler, Anja Seger, Christopher Bohr

**Affiliations:** ^1^Division for Phoniatrics and Pediatric Audiology, Department of Otorhinolaryngology, Head and Neck Surgery, Medical School, FAU-Erlangen-Nürnberg, Bohlenplatz 21, 91054 Erlangen, Germany; ^2^Department of Communication Sciences and Disorders, Louisiana State University, 64 Hatcher Hall, Baton Rouge, LA 70803, USA; ^3^Section of Experimental Oncology and Nanomedicine (SEON), Department of Otorhinolaryngology, Head and Neck Surgery, Medical School, FAU-Erlangen-Nürnberg, Glückstrasse 10a, 91054 Erlangen, Germany

## Abstract

The current use of laryngeal high-speed videoendoscopy in clinic settings involves subjective visual assessment of vocal fold vibratory characteristics. However, objective quantification of vocal fold vibrations for evidence-based diagnosis and therapy is desired, and objective parameters assessing laryngeal dynamics have therefore been suggested. This study investigated the sensitivity of the objective parameters and their dependence on recording frame rate. A total of 300 endoscopic high-speed videos with recording frame rates between 1000 and 15 000 fps were analyzed for a vocally healthy female subject during sustained phonation. Twenty parameters, representing laryngeal dynamics, were computed. Four different parameter characteristics were found: parameters showing no change with increasing frame rate; parameters changing up to a certain frame rate, but then remaining constant; parameters remaining constant within a particular range of recording frame rates; and parameters changing with nearly every frame rate. The results suggest that (1) parameter values are influenced by recording frame rates and different parameters have varying sensitivities to recording frame rate; (2) normative values should be determined based on recording frame rates; and (3) the typically used recording frame rate of 4000 fps seems to be too low to distinguish accurately certain characteristics of the human phonation process in detail.

## 1. Introduction

The human voice originates in the larynx from the oscillation of the two opposing vocal folds, Figure 2. Anatomical changes of the vocal folds and functional disorders can lead to changes in voice quality [1]. Endoscopic investigation of laryngeal structures allows direct visualization of vocal folds and their function during phonation. Currently, videostroboscopy [2] is the gold standard for investigating vocal fold function in clinical settings whereas high-speed videokymography and laryngeal high-speed videoendoscopy (HSV) are mainly used as supporting and additional imaging tools [3]. However, stroboscopy only allows the visualization of periodic vocal fold oscillations [3]. Stroboscopy is triggered by the vocal fundamental frequency and is therefore not applicable for smeared and not well-defined fundamental frequencies that are present in aperiodic vocal fold dynamics often associated with voice disorders [4]. In contrast, HSV permits the visual capture of the complete inter- and intracyclic behavior of the vocal fold dynamics [5–7]. Hence irregular and aperiodic vocal fold dynamics and also, for example, mucosal wave propagation can be visually observed and analyzed [8, 9]. This is due to the temporal recording rate of HSV, typically 2, 4, and 8 kfps, being much higher than the fundamental vocal fold oscillation frequency of 100–300 Hz for normal human phonation [10, 11]. So far, HSV recordings have been mainly analyzed subjectively using perceptual evaluation of the data [11], which, depending on the study, seems to yield no advantages [12], minor to mild advantages [9, 13], or significant advantages [14] compared with stroboscopy. Consequently, these contradictory results do not justify the high acquisition costs of HSV. To establish HSV in daily clinical routine and actually to obtain advantages by applying HSV, image processing algorithms were developed to allow the computation of objective and quantitative measurements for HSV [15–17]. Based on these image processing algorithms, the first user-friendly software tools were suggested and applied in several studies [6, 18–20]. The basis for a quantitative HSV analysis is the segmentation of the glottis [15], namely, the area between the two vocal folds, Figure 2. The segmentation of the glottis for all HSV frames yields the glottis area change over time and is called the glottal area waveform (GAW) [19], Figure 3(a). Using the GAW, objective and quantitative parameters can be computed describing the phonation process at the vocal fold level and providing information on periodicity and asymmetry characteristics of the vocal fold dynamics [21].

However, HSV is not yet established in clinical routine owing to several restrictive factors. Compared with stroboscopy, HSV hardware is rather expensive [22], there are no official guidelines for HSV footage analysis, and the additional benefit for daily clinical routine is still under discussion [4, 10, 11]. A further reason, despite existing studies with several hundred subjects [23], is the lack of normative parameter values and intervals, which are needed to determine the severity of pathological voice production. Also, studies have shown that there are significant parameter value differences between gender [23] and age groups [20, 24], complicating the definition of normative and clinically relevant data.

The HSV tool and its data can also influence the parameters of vocal fold vibratory function. These factors, which are expected to influence HSV parameters but have not been investigated thoroughly, include the recording frame rate (fps), the spatial resolution (pixels), and the analyzed interval length (i.e., the number of vocal fold oscillation cycles considered). Hence, in this study we investigated the influence of the HSV recording frame rate on quantitative GAW parameters, since many different recording rates have been used in existing studies. Popular HSV recording frame rates are 2 kfps [7], 4 kfps [6], and 8 kfps [25]. Further, it was shown that too low HSV recording rates might also restrict subjective HSV analysis [9]. This study suggested, by analyzing healthy females, that 2 kfps might be insufficient to judge subjectively certain vocal fold dynamic characteristics at oscillation frequencies above 200 Hz. From the technical point of view, current high-end high-speed cameras enable frame rates of several 100 kfps to be used. The latest studies in which these cameras were applied achieved recording frame rates of up to 20 kfps and could record and analyze high soprano voices with frequencies up to 1568 Hz [26]. However, a disadvantage of very high recording frame rates is the potential decrease in spatial resolution. The camera model used in our work (Photron Fastcam SA1.1, Photron USA Inc., 2014), for example, permits a frame rate of 5.4 kfps at a spatial resolution of 1024 × 1024 pixels. For 20 kfps, the spatial resolution is reduced to 512 × 512 pixels and for 75 kfps to 512 × 128 pixels.

In summary, HSV has the potential to support and supplant the clinical diagnosis of voice disorders and vocal fold vibratory dysfunction and objectively document therapy outcome. However, there is a need to establish normative data for HSV parameters to enhance the clinical importance and significance in clinical practice of this powerful imaging technique. In order to achieve this, one of the first aspects to establish is the influence of HSV recording frame rate on HSV parameters. To the best of our knowledge, there have been no studies on the investigation of the influence of the HSV recording frame rate on computed quantitative HSV parameters. However, as reported recently [20], there is a need to analyze the behavior and stability of HSV parameters with varying HSV recording rates. Hence, this aspect was the focus of the present study, in which we aimed to increase the awareness of this problem and to encourage a standardized computation of HSV parameters.

The goal of this study was not to acquire normative values based on many subjects but to demonstrate the sensitivity of objective HSV parameters to the recording frame rate. For this purpose, 300 HSV recordings for one subject with varying frame rates were analyzed.

## 2. Materials and Methods

Ethical approval was obtained from the University of Erlangen-Nürnberg (number 290_13B). The participant gave informed, written consent prior to participation and this consent procedure was approved by the local committee. Experiments were performed in accordance with the Declaration of Helsinki (1964).

### 2.1. Data

HSV recordings were performed for a female subject during sustained phonation of the vowel /i/ at a comfortable pitch and loudness level for the participant. A 70° endoscope was coupled to a gray-scale high-speed camera (Photron FASTCAM SA1.1 Model 675K-M1). HSV recordings were performed at 15 kfps resolution in time at a spatial resolution of 768 × 512 pixels, being superior to current clinical HSV systems [20, 21]. At the time of the experiment, the female participant was vocally healthy, a nonsmoker, and 45 years old. A total of 24 HSV recordings were performed. The averaged phonatory fundamental frequency *F*_0_ was 176 ± 11 Hz with sound pressure levels around 74 dB. Of the original 24 recordings, four were excluded owing to a lack of the desired number of glottis cycles, bad image quality, and full vocal fold length not being present. All HSV data analyzed had at least 106 oscillation cycles during sustained phonation to be segmented and analyzed. We chose to analyze 106 oscillation cycles since The National Center for Voice and Speech recommended (for acoustic signals) the analysis of a minimum sequence length of 100 oscillation cycles [27]; another study even proposed at least 110 cycles [28]. This yielded 20 original HSV recordings for further analysis.

### 2.2. Preprocessing: Glottis Segmentation

First, the frame rate of the 20 HSV movies was reduced stepwise in 1 kfps steps from 15 to 1 kfps. This yielded 15 movies, from 1 to 15 kfps, for each original HSV recording, resulting in a total of 300 HSV sample movies for further analysis. Next, the 15 movie samples from each original recording were image processed with identical segmentation parameters so as to eliminate influences from the image processing algorithms. For glottis segmentation, our in-house developed software* Glottis Analysis Tools (GAT)* was applied, providing the GAW over an interval of 106 oscillation cycles for each movie. From the GAW (Figure 3(a)), the objective and quantitative parameters were computed as described in the following.

### 2.3. HSV Parameters Computed from the GAW

A total of 20 parameters were computed and analyzed. The parameters considered were chosen because (1) they are already frequently applied in daily clinical routine and in other studies, (2) they reflect different characteristics of the vocal folds and glottis dynamics, and (3) they are computed on different spaces (spectral and time space of the GAW). Although there are certainly more objective parameters being used in HSV analysis [8, 23, 29], the consideration of all possible HSV parameters would go far beyond one study. The primary goal of our study was to determine if there is a frame rate dependence among the HSV parameters. The 20 parameters investigated in this study are listed in Table 1 and they were used to examine (A) glottal dynamic characteristics, (B) glottal perturbation characteristics, and (C) glottal noise components.

### 2.4. Statistical Analysis

For statistical analysis, IBM SPSS Statistics Version 21 was used. All parameters were tested for normal distribution (Shapiro-Wilk test and Lilliefors modification of Kolmogorov-Smirnov test). The significance level was set to 5% (*p* < 0.05). The tests showed that none of the parameters was normally distributed (*p* < 0.05). Therefore, nonparametric tests were used for further analysis.

The goal of the statistical analysis was to establish whether the parameter values change significantly with increase in recording rate from 1 to 15 kfps. For each parameter, it was checked if the 15 sample sets (recording rates 1–15 kfps) showed significant differences, indicating a dependence on the recording frame rate. Therefore, the Friedman test for comparing more than two dependent samples (here 15 samples) was applied. One sample consisted of the parameter values of all 20 movies for the corresponding recording rate (1–15 kfps). This means that 15 dependent samples (number of different recording rates) containing 20 values (number of HSV recordings) were compared. Except for two parameters (PA, OQ), the Friedman test showed that there are statistically significant differences for at least two samples (i.e., recording rates). However, it does not indicate which samples are different. Therefore, for further analysis, the Wilcoxon test for two dependent samples was applied. This means that, for each of the remaining 18 parameters, the sample with 1 kfps was tested against each higher frame rate from 2 to 15 kfps, the sample with 2 kfps was tested against the samples from 3 to 15 kfps, and so on. Hence, each parameter was tested 14 times for each frame rate. Therefore, a Bonferroni correction of the significance level *p* was applied, yielding the new significance level *p* = 0.05/14 = 0.0036.

## 3. Results

The behavior of the GAW parameters with respect to the HSV recording rate resulted in four main categories:

- Group 1: parameters remain stable; that is, the parameter values over the 15 frame rate samples are equal and hence independent of the recording rate.
- Group 2: parameters remain unstable only until a certain frame rate is reached, and after that frame rate the parameter values are rate stable up to 15 kfps.
- Group 3: parameters are stable within a certain HSV recording range interval (at least four recording rates) and change at lower and higher recording rates.
- Group 4: parameters are highly dependent on HSV recording rate. These parameters change their values between each recording rate and are only stable for not more than three different HSV recording rates.

**Figure 1:**
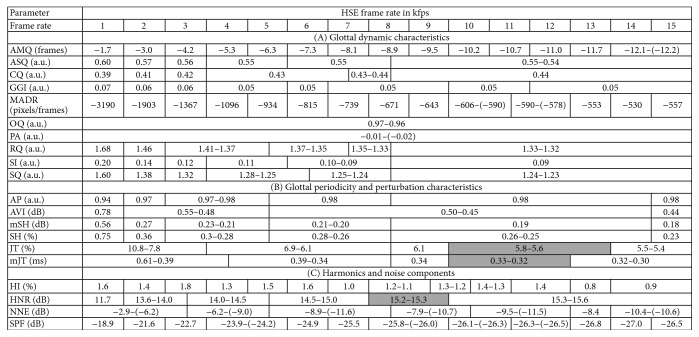
Computed mean parameter values for the different recording frame rates. For statistically not significantly different *p* values (*p* > 0.0036), the values are merged and the parameter value range is given. Gray-shaded areas mean that there is no statistical difference from the left and right recording frame rate intervals.

**Figure 2:**
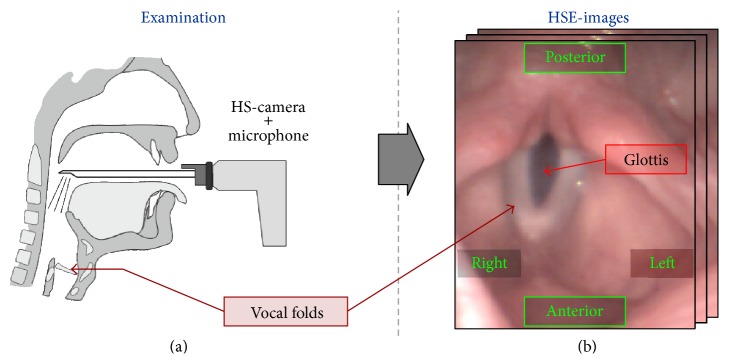
HSV recording setup and view of the vocal folds as seen through the camera. In (b), the dark glottis between the two vocal folds can be seen.

**Figure 3:**
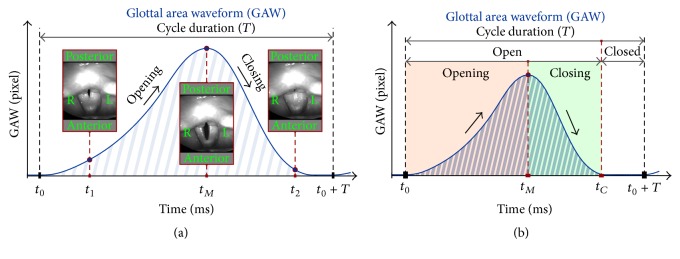
(a) Glottal area waveform (GAW) and the subdivision of the different oscillation states for computing the GAW parameters. (b) Definitions of GAW conditions used for parameter computation.

An overview of the computed mean values of the parameters for the corresponding recording rates is given in Figure 1. Recording rates showing no statistical differences are merged and the minimal and maximal values of corresponding means are given. Gray-intervals (JT, mJT, and HNR) indicate that these intervals do not show statistically significant differences from their left and right neighboring intervals. However, the intervals on the left and right borders of the gray-shaded interval are statistically different. Owing to space limitations, we have not given *p* values, as a total of 105 × 20 = 2100 *p* values would have had to be reported.

*(A) Glottal Dynamic Characteristics*. AMQ and MADR are highly recording rate dependent* (group 4)*. These two parameters are stable over at most two HSV recording rates. The five parameters ASQ, CQ, RQ, SI, and SQ exhibit no changes from 8 kfps and belong to* group 2*. GGI does not change after 12 kfps* (group 2)*. The two parameters OQ and PA seem to be independent of the recording rate, since they do not change at all and hence belong to* group 1*. 

*(B) Glottal Periodicity and Perturbation Characteristics*. All four parameters reflecting amplitude perturbation are in* group 3*. Whereas AP, mSH, and SH were stable between recording rates of 8 and 14 kfps, AVI did not change between 5 and 14 kfps. In contrast, both oscillation time perturbation measures JT and mJT are found in* group 2*. These two parameters did not change any further from a recording rate of 10 kfps. 

*(C) Harmonics and Noise Components*. These parameters, except HNR, are computed over the frequency space of the GAW and change for nearly every recording frame rate. Whereas HI, NNE, and SPF belong to* group 4*, HNR shows stability for 8 kfps and higher* (group 2)*.

Considering the stability of the parameters, the following can be observed. Two (10%) parameters (OQ, PA) are independent of the recording frequency* (group 1)*. Nine parameters (45%) (ASQ, CQ, GGI, RQ, SI, SQ, JT, mJT, and HNR) do not change any further after a certain recording rate* (group 2)*, that is, after 8, 10, or 12 kfps. Four parameters (20%) (AP, AVI, mSH, and SH) are stable over at least four frequency intervals (one parameter 5–14 kfps and three parameters 8–14 kfps) but change again at the highest recording rate* (group 3)*. Five parameters (25%) (AMQ, MADR, HI, NNE, and SPF) are highly dependent on the recording frequency and change for almost every recording rate* (group 4)*. In particular, parameters computed in the frequency space (HI, NNE, and SPF) of the GAW seem to be highly dependent on the HSV recording rate.

**Figure 4:**
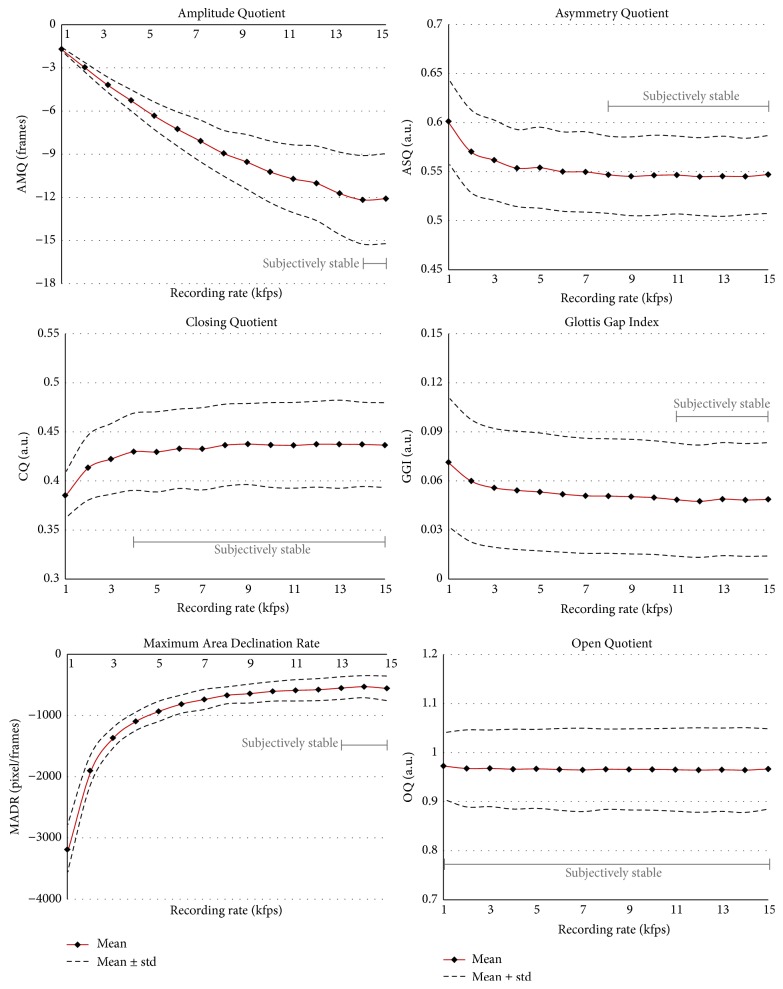
The first six parameters representing glottal dynamic characteristics. Means and standard deviations are given. The subjective rating of stable values over HSV recording frame rates is indicated.

**Figure 5 fig5:**
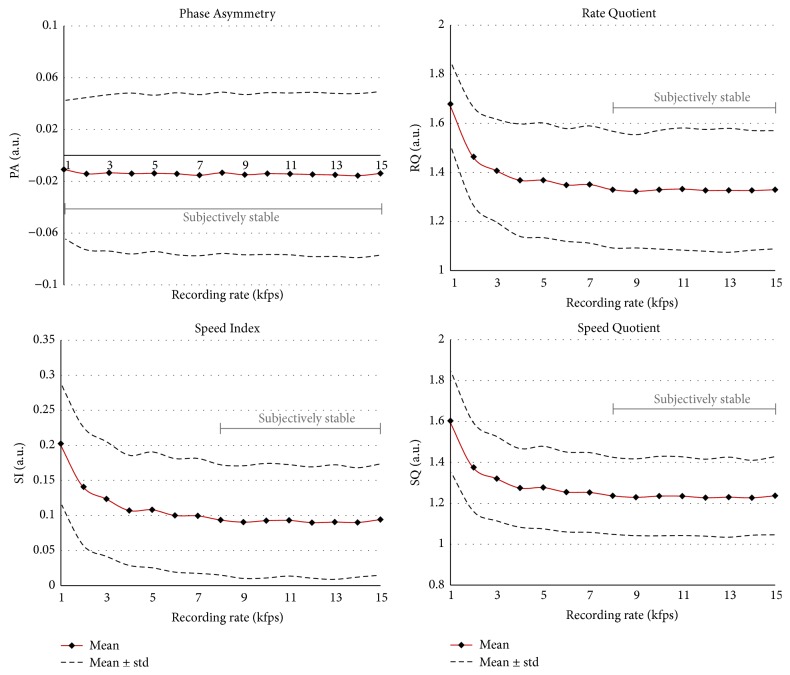
The other four parameters representing glottal dynamic characteristics. Means and standard deviations are given. The subjective rating of stable values over HSV recording frame rates is indicated.

**Figure 6 fig6:**
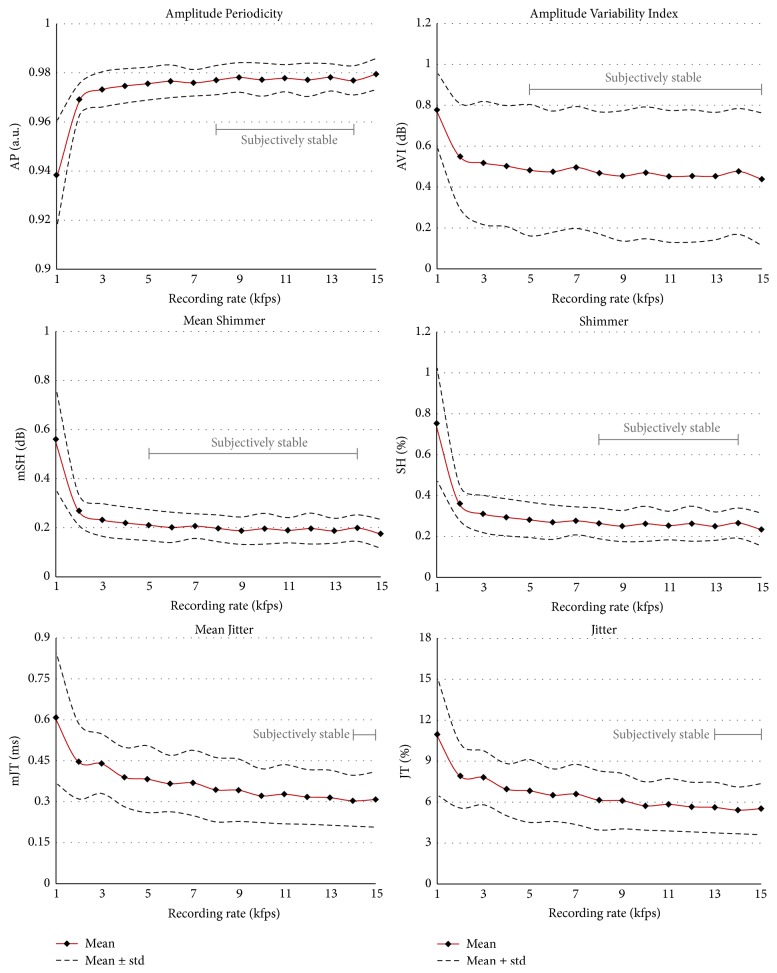
The six parameters representing glottal perturbation. Means and standard deviations are given. The subjective rating of stable values over HSV recording frame rates is indicated.

**Figure 7:**
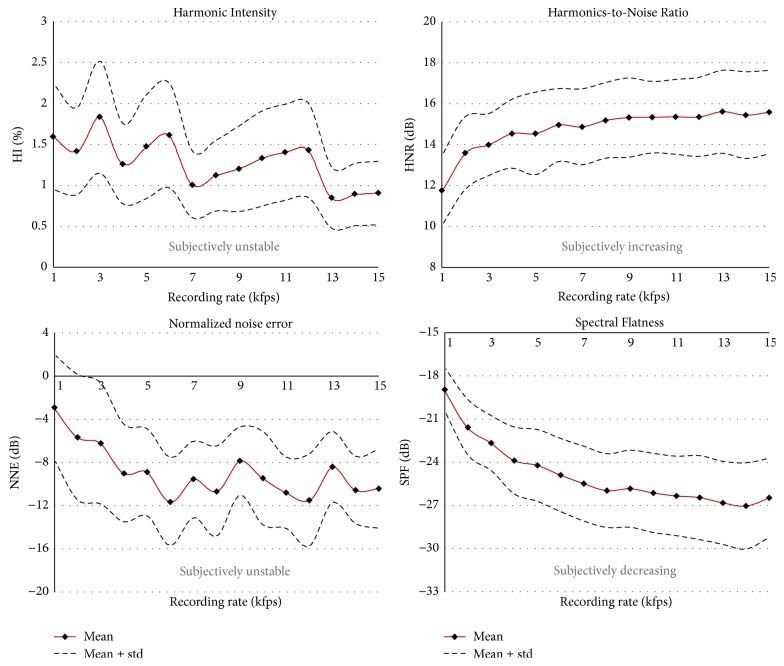
Parameters representing glottal harmonic and noise components. The parameters HI, NNE, and SPF are computed on the spectral space of the GAW. Means and standard deviations are given. The obvious, subjectively and objectively determined, instability, and the dependence on the HSV recording rates suggest that HI, NNE, and SPF are not suitable for evaluating the GAW signal.

To give a graphical impression of the behavior of the parameters with respect to the HSV recording rate, the averaged means and corresponding standard deviations for each recording rate are given in Figures 4 [Fig fig5] [Fig fig6]–7. Additionally, the behavior of the parameters with respect to the HSV recording rates was subjectively rated by four experts. The experts were asked to define intervals for the parameters where they subjectively and visually assume the parameters to be stable and constant. All raters were in complete agreement for the chosen and indicated intervals as illustrated in Figures 4 [Fig fig5] [Fig fig6]–7. For 12 out of the 20 parameters, the experts confirmed the statistical results and rated identically. For five parameters (CQ, GGI, MADR, AVI, and mSH), subjective evaluation of the stability by the raters showed that some of the statistically different intervals did not appear to change visually, whereas for three parameters (mJT, JT, and HNR) the experts visually evaluated changes where the statistics found no significant differences. However, these discrepancies were small and the subjective ratings closely reflected the statistical results as presented in Figure 1.

## 4. Discussion

The analysis on the dependence of HSV parameters on the HSV recording frame rate showed that 90% of the considered parameters change with frame rate. However, for the two parameters that did not show such a dependence this result will have to be confirmed in a larger set. The statistical results were mostly visually confirmed by the subjective perception of four experts.

In the following, the computed parameter values are discussed and compared with those obtained in previous investigations. If not explicitly noted, we refer to studies dealing with healthy female subjects investigated by HSV. When first mentioned that the configuration and details of each study are given in a table. The tables include means and standard deviations of the parameters, age and number of subjects, phonated tone, and fundamental frequency. The camera settings (recording frame rate and pixel resolution) and considered signal length are also given.

The purpose of comparing our parameter values with other study results was not to propose normative values but primarily to show that the variance of computed parameter values is often based on various applied HSV settings limiting the significance of computed absolute parameter values and also reducing the comparability between different studies. This emphasizes the need for detailed documentation within studies, but more important the need for well-defined HSV recording guidelines and normative parameter values based on potential influencing factors such as HSV recording frequency, spatial HSV resolution, age, and gender. This will then permit a better separation between healthy and disordered voice production and therefore enhance the clinical usefulness of HSV in the future.

*(A) Glottal Dynamic Characteristics*. The* Amplitude Quotient (AMQ)* belongs to* group 4*. In other words, AMQ values reach a stable state at 14 and 15 kfps. From 1 to 13 kfps the samples are found to be statistically different. The values for AMQ decrease continuously with increase in recording rates from –1.7 to –12.2. The difference of AMQ at 4 kfps (–5.3) compared with the value for 15 kfps (–12.2) is high at 57%. Compared with other studies (Table 2), our AMQ values at 4 kfps are slightly lower. The results suggest that AMQ is highly influenced by the recording rate and that preferably very high recording rates should be used to achieve reliable results.

The* Asymmetry Quotient (ASQ)* belongs to* group 2* and does not change after a recording rate of 8 kfps (ASQ = 0.55–0.54). These values are similar to those of 0.59 ± 0.05 [41] and 0.52 ± 0.06 [21] obtained using 4 kfps. The changes in ASQ seem to be minor, with a maximum of 0.60 at 1 kfps, even though the differences are statistically significant. For higher recording rates, the ASQ values decrease continuously but to a small extent. For example, between 4 and 15 kfps there is only a difference in the second decimal place. Hence, it appears that a 4 kfps recording rate might be sufficient for ASQ computation.

For the* Closing Quotient (CQ)*, the values do not change significantly from 8 kfps* (group 2)*. A previous study computed CQ at 0.45 ± 0.07 [21], which is very similar to our value at 15 kfps (0.44), although they only used a recording rate of 4 kfps. Also, the presented values here at 4 kfps are in the same range (0.43). Another study reported a lower CQ value of 0.34 [42]; there the parameter was called closing phase, Table 3.

Mehta et al. (2011) computed for seven healthy subjects (men and women) a lower CQ (0.26 ± 0.08) [43]. This CQ value is also below computed values for solely male groups presented previously: 0.40 ± 0.09 [46] and 0.38 ± 0.11 [41].

In summary, all CQ mean values in these studies are between 0.26 and 0.45 and are in the range reported by Holmberg et al. (1988) for healthy women [32]: 0.21–0.48. However, they computed their CQs from the glottal flow (i.e., inverse filtered) and not from the HSV-derived GAW.

The* Glottis Gap Index (GGI)* does not change after 12 kfps and belongs to* group 2*. Overall, the values from GGI change only slightly from 0.07 to 0.05, although showing statistically significant differences. However, the clinical significance of this finding needs to be further determined. The values confirm previous measurements for healthy female subjects [19]: 0.054 ± 0.072, Table 4.

The* Maximum Area Declination Rate (MADR)* changes significantly for almost all recording rates* (group 4)*. It increases continuously with increase in recording rate from –3190 (1 kfps) to –530 (14 kfps). This behavior is not surprising, since MADR was computed for pixels/frames and hence naturally decreases with increasing HSV recording rates. However, it seems that MADR stabilizes at 13 kfps and above; that is, the values remain in the range –530–(–557). Patel et al. (2014) [41] also used a 70° endoscope and computed similar MADR values of–1011 ± 290 at a comparable recording rate of 4 kfps (here –1096 ± 150). The lower value of –628 ± 186 reported by Bohr et al. (2013) [21] at the same HSV recording rate of 4 kfps may be explained by the lower spatial resolution and that they used a 90° endoscope. The 90° endoscope has a larger distance to the glottis than the 70° endoscope and the glottis area size is naturally smaller in the HSE images. This, in combination with the reduced pixel resolution, reduces the absolute number of pixels in the glottis and hence the pixel changes and therefore the absolute MADR values.

The* Open Quotient (OQ)* remains constant at 0.97–0.96 for all recording frame rates and is therefore independent of it* (group 1)*. A previous study reported similar values (0.95 ± 0.09) [21] whereas another study reported slightly lower values (0.86 ± 0.17) [41].

Significantly lower values were found in other studies: namely, 0.68 ± 0.12 [42], 0.64 ± 0.10 [33], and 0.57 ± 0.18 [43]. Ahmad et al. (2012) [7] also computed lower OQ values (0.68–0.78), which may be explained by the fact that they defined the glottis as fully closed when the opening area of the glottis was less than 5% of the maximum opening area. Since a posterior glottal chink is common in females, Inwald et al. (2011) [23] found that 47.9% of healthy females exhibited a small posterior glottal chink and Ahmad et al. may have overestimated the actual “fully closed” time for females.

Similarly, Ikuma et al. (2014) [44] defined the glottis as being closed when the actual glottis area was below 1% of the maximum glottal area. They computed the OQ for a postsurgery female and male group as 0.91 ± 0.11, that is, closer to our values.

We conclude that for OQ it should be defined whether such thresholds will be used and at what cut-off level. Further, depending on the image processing applied, algorithms might consider certain pixels as glottis or not at the state of a nearly closed glottis, which in consequence yields different results for OQ.

For males [6] and children [20], this issue seems to be less important as the posterior glottal chink occurs less commonly, resulting in lower OQ values, Table 5. However, large variations have been reported for OQ. Holmberg et al. (1988) [32] computed the ranges 0.56–0.95 for normal females and 0.46–0.77 for normal males using the inverse filtered airflow signal. Hence, it may be difficult to give common valid normative values that might differentiate normal from disordered voices.

The* Phase Asymmetry (PA)* seems to be equal for all recording frame rates considered* (group 1)*. The computed means in our study vary between –0.02 and –0.01, reflecting high dynamic left–right symmetry. Zero corresponds to perfect left-right symmetry; that is, the left and right GAW, separated by the glottis axis, reach their maximal area at exactly the same time. Bohr et al. (2013) [21] computed similar values at –0.02 ± 0.07 (they called this parameter “Glottis Phase”).

The* Rate Quotient (RQ)* does not change significantly from 8 kfps* (group 2)*. There, the means are between 1.33 and 1.32. In contrast, Patel et al. (2014) [41] computed much higher values in the range 2.04 ± 0.78. These values are also significantly higher than comparable RQ values at 4 kfps in our study (1.41–1.37). One explanation might be the higher phonation fundamental frequency of the subjects in Patel et al.'s study compared with ours.

In summary, the RQ values for the typical clinical recording rate (4 kfps) and the highest recording rate differ by only 6%, showing a fairly stable RQ despite statistically significant differences.

The* Speed Index (SI)* does not change significantly from 8 kfps and remains at a level of 0.09* (group 2)*. Ikuma et al. (2014) [44] reported similar values for their cohort (i.e., postsurgery status of a mixed female and male group of eight subjects) at 0.10 ± 0.07 for a recording frame rate of 2 kfps. The equivalent value in our study at 2 kfps is similar at 0.14. A higher value of 0.18 was reported elsewhere [41]. Again, this might be explained by the increased mean phonatory fundamental frequency in their subjects. They reported a mean of 251 Hz [41], which is much higher than in our study (176 Hz).

The values for SI in our study show fairly large differences with increase in recording rate (i.e., 22% between 4 and 8 kfps). Hence, we suggest that a frame rate of 8 kfps and higher should be used to compute SI.

The* Speed Quotient (SQ)* does not show significant differences from 8 kfps (1.24–1.23). Also, the difference from 4 kfps (1.28) at 4% is small. In particular, the values of Bohr et al. (2013) [21], with a mean of 1.17 ± 0.33, are very similar, although they analyzed a group of young women (age around 20 years). Mehta et al. (2011) [43] also reported similar results for SQ (1.15 ± 0.52) whereas Kunduk et al. (2010) [33] computed slightly lower values of 0.99 ± 0.13. Apart from the statistical analysis, it can be stated that SQ hardly changes between 3 and 15 kfps. However, below 3 kfps, SQ is significantly higher. Hence, we suggest that, for computing SQ, a recording rate of at least 4 kfps should be used. Interestingly, Holmberg et al. (1988) [32], for the inverse filtered airflow signal, reported much higher values in the range 1.19–2.33. However, these higher values may be explained by inertance effects of the sub- and supraglottal air columns and their interaction during opening and closing of the glottis.

*(B) Glottal Periodicity and Perturbation Characteristics*. The* Amplitude Periodicity (AP)* does not show changes between 8 and 14 kfps* (group 3)*. In summary, only the value for a recording rate of 1 kfps (0.94) is obviously lower than the others (0.97–0.98). Despite the statistical differences for the recording rates, it can be assumed that a 2 kfps recording rate might be sufficient. Our values are confirmed by previous work in which AP values of 0.977 ± 0.009 were reported [19].

The* Amplitude Variability Index (AVI)* does not change statistically between 5 and 14 kfps* (group 3)*. Interestingly, at 15 kfps statistically significant differences occur. The mean values for recording rates of 2–4 kfps are slightly higher but basically in a similar range.

The* Mean Shimmer (mSH)* does not change statistically between 8 and 14 kfps* (group 3)*. Between 2 and 15 kfps the values are fairly similar (0.27–0.18). Only for 1 kfps is the mSH value greatly increased (0.56). Bohr et al. (2013) [21] computed values of 0.19 ± 0.08, which are in the same range as in our study for 3–15 kfps.

For* Shimmer (SH)*, as for mSH, the values do not change statistically between 8 and 14 kfps* (group 3)*. The value at 15 kfps is again statistically different. However, the values change only slightly from 3 kfps (0.31–0.23%), being in the same range as those reported by Bohr et al. (2013) [21] of 0.29 ± 0.13%. However, other studies reported higher values. Yan et al. (2005) [45] computed 2.1% for 2 kfps, although they did not give the gender or age of the subjects, Table 6. Ahmad et al. (2012) [7] computed for young healthy females values of 1.5–7.2% at 2 kfps. For older healthy women a high value of 6.2% was also reported [24]. The above three studies all used a low HSV recording rate of 2 kfps, which might be a reason for their different and high values. Also, the factors spatial resolution and number of considered oscillation cycles might have an influence on SH.

*The Jitter (JT)* values do not change from 10 kfps (5.8–5.4%)* (group 2)*. However, the values for lower recording rates (10.8–7.8%) are almost twice as high. The value of 5.7 ± 3.0% reported by Bohr et al. (2013) [21] is in the range of values computed in our study for 10 kfps and higher. The comparable value at 4 kfps at 6.9% is in the range of Bohr et al.

Ahmad et al. (2012) reported lower JT values for older (3.8%) and younger (0.9–3.6%) females [7, 24]. Yan et al. (2005) [45] also computed a lower value of 1.4%. Similarly to SH, further influencing factors seem to be present in computing JT.

The* Mean Jitter (mJT)* does not change significantly for 10–15 kfps (0.33–0.30 ms)* (group 2)*. The decrease of 51% over frame rates from 1 to 15 kfps indicates a strong dependence. However, from 4 kfps (0.39 ms) mJT seems to stabilize. Bohr et al. (2013) [21] reported for 4 kfps a mJT of 0.2 ms, being 33% lower than the value computed here at 15 kfps (0.30 ms). The difference (49%) from the corresponding value in our study at 4 kfps (0.39 ms) is even higher. As for SH and JT, it may not only be the HSV recording rate that influences the computation of mJT.

*(C) Harmonics: Noise Signal Components*. The* Harmonic Intensity (HI)*, as found statistically, changes over the entire range of HSV recording rates. Further, as seen in Figure 7, HI does not exhibit a definite trend but seems highly unstable. This unstable behavior suggests that HI is not suitable for GAW analysis and therefore should not be considered in future work. No studies of HI applied to the GAW signal were found in the literature.

For the* Harmonics-to-Noise Ratio (HNR)*, statistical analysis showed two stable intervals: 5–9 and 8–15 kfps* (group 2)*. A previous study computed HNR values of 14.9 ± 5.9 dB for 4 kfps [21], covering the HNR values over all our recording rates. Significantly higher values were reported by Ikuma et al. (2014) [44] for postsurgical subjects (37.6 ± 2.6 dB) and also for healthy subjects (>32.05 dB) [47]. However, they used a low HSV recording rate (2 kfps) and did not provide age or gender details. The HNR values in the latter two studies are also much higher than those computed for women from the acoustic signal (24.32 ± 4.25 dB) [48]. Owing to the higher recording rate of acoustic signals (44 kHz), one would actually expect better HNR values for the acoustic signal. Potentially, the deviation from the earlier studies [44, 47] is based on a different HNR computation formula.

For the* Normalized Noise Energy (NNE)*, statistically the values are significantly different for almost all recording rates* (group 4)*, as also confirmed by visual inspection, Figure 7. Between the lowest and highest recording rates there is a difference of 366%, indicating a very high dependence on the recording rate. Bohr et al. (2013) [21] computed at 4 kfps a mean NNE of –10.4 ± 7.2 dB, similar to the values at 14 and 15 kfps in our study. However, the standard deviation in their study is large and includes NNE values between –3.2 and –17.6 dB. This interval coincides with the values computed in this study between 1 and 15 kfps. These extreme variations indicate that NNE values computed on the GAW signal might not be clinically applicable.

The* Spectral Flatness (SPF)* is found to be statistically and subjectively unstable* (group 4)*, Figure 7. In summary, the values change from –18.9 to –26.5, representing a difference of 29%. This unstable behavior, as already seen for the three previous parameters computed on the spectral space, does not favor clinical applications in the future.

## 5. Recommendation for HSV Recording Rates

Taking a closer look at the typical recording frame rate of 4 kfps used in clinical HSV settings, it can be seen that all parameters, except OQ and PA (*group 1*), change at higher recording rates, Figure 1. These findings concur with considerations by Patel et al. (2016) [20] and it raises the question of whether for these parameters the computed values at 4 kfps reflect the actual vibratory vocal fold characteristics or whether they are only a more or less accurate approximation of the real vocal fold dynamics. However, the relative small parameter changes between 4 and 15 kfps for the parameters in* (A) glottal dynamic characteristics* (except AMQ and MADR) and* (B) glottal periodicity and perturbation characteristics* (Figures 4 [Fig fig5]–6) justify the application of a recording rate of 4 kfps in clinical studies and daily clinical routine.

Nevertheless, it should be stated that, from a recording rate of 8 kfps, 11 parameters (55%) do not change statistically or show only negligible differences at higher recording rates. Hence, in scientific studies where the exact vocal fold characteristics are of interest, we recommend a recording rate of at least 8 kfps, and higher if possible. In other words, based on the fundamental frequencies of the subject in this study being close to 200 Hz, the results suggest that the frame rate of the camera should be 40 times higher than the vocal fold oscillations to achieve stable values for certain dynamic parameters.

## 6. Limitations

Several limitations are evident in this study. The recordings for only one subject were analyzed. However, for each frame rate from 1000 to 15 000 fps for 20 initial recordings resulting in a total of 300 HSV data sets were analyzed to support the findings of this study. Every effort was made to ensure similarity in the endoscope-vocal fold distance, and the effects of varying endoscope-vocal fold distance on the parameters investigated here need to be considered in a further study. In addition, the HSV recordings were carried out in one session; hence, it is not known if fatigue factors were influencing the variability of HSV parameter values.

The recordings of only one subject were analyzed, which allows the conclusion that parameters are dependent on the HSV recording rate if they show statistically significant differences for different recording rates, but this does not enable us to conclude definitely and universally that the parameters that did not show differences will do so in a large cohort. Also, the interval boundaries where dependences on the recording rate were found and the corresponding parameter values (Figure 1) cannot be generalized based on one subject. Hence, an extended study, considering age and gender, will have to be performed to confirm the observed behavior with respect to different HSV recording frame rates.

Further, the influence of the spatial HSV resolution will have to be investigated in the future. The various studies discussed here exhibit a wide range of spatial HSV resolution that might also be responsible for the different computed parameter values.

The number of oscillation cycles considered might also play a role in HSV parameter variability, as seen before in acoustic signals [49]; for example, the studies discussed here reported between around 4 and 110 oscillation cycles.

The influence of the applied segmentation techniques on the GAW and hence on the absolute parameter values is of interest and will have to be analyzed in the future. Depending on the HSV image quality, different image processing algorithms may yield slightly different segmentation results and hence GAW curves. This directly transfers to the computed GAW parameters. In our study, we eliminated this error source by eliminating HSV movies with low image quality.

## 7. Conclusion

It was shown that the majority (90%) of the investigated parameters are dependent on the HSV recording rate. The influence of the recording rate on HSV parameters varied differently and clearly. Hence, this is a factor not to be neglected during quantitative HSV analysis. To overcome this issue and to bring HSV into clinical routine, we suggest further investigations of potential influencing factors such as HSV recording rate, HSV spatial resolution, number of considered vocal fold oscillations, and gender and age of the subjects. From there, normative tables, if needed separated for these factors, could be introduced. Finally, this study showed that the commonly used HSV recording rate of 4 kfps may be insufficient to distinguish the vibratory vocal fold characteristics in detail.

## Figures and Tables

**Table 1:** The parameters analyzed, with units, value ranges, and descriptions. Definitions of the GAW conditions are illustrated in Figure 3.

Parameter (abbreviation) & reference	Units	Range	Description/formula
(A) Glottal dynamic characteristics			

Amplitude Quotient (AMQ) [30]	Frames	<0	Glottal area (GA) dynamic range/MADR
Asymmetry Quotient (ASQ) [31]	—	[0; 1)	SQ/(SQ + 1)
Closing Quotient (CQ) [32]	—	[0,1)	Closing/*T*
Glottis Gap Index (GGI) [19, 33]	—	[0; 1]	Min (GA)/max (GA)
Maximum Area Declination Rate (MADR) [34]	Pixels/frames	<0	Value of negative peak in the 1st derivative of GAW, that is, max. GAW closing velocity
Open Quotient (OQ) [35]	—	[0; 1]	Open/*T*
Phase Asymmetry (PA) [36]	—	(−1; 1)	(*t*_*M*_ (Left) − *t*_*M*_ (Right))/*T*
Rate Quotient (RQ) [35]	—	>0	(Closed + opening)/closing
Speed Index (SI) [35]	—	(−1; 1)	(SQ − 1)/(SQ + 1)
Speed Quotient (SQ) [35]	—	≥0	Opening/closing

(B) Glottal perturbation characteristics			

(B1) Amplitude stability: these parameters reflect the periodicity of the GAW area			
Amplitude Periodicity (AP) [36]	—	[0; 1]	Ratio (min/max) of the GA dynamic ranges of 2 consecutive GAW cycles
Amplitude Variability Index (AVI) [35]	dB	(−*∞*; +*∞*)	That is, dB-scaled coefficient of variation applied to GA dynamic ranges of all GAW cycles
Mean Shimmer (mSH) [35]	dB	≥0	Mean dB-scaled difference between the GA dynamic ranges of 2 consecutive GAW cycles
Shimmer (SH) [37]	%	[0; 100]	Ratio between mSH and mean dB-scaled GA dynamic range over all GAW cycles

(B2) Period length stability: these parameters reflect the time periodicity of the GAW			
Jitter (JT) [37]	%	[0; 100]	Ratio between mJT and mean duration over all GAW cycles
Mean Jitter (mJT) [37]	ms	≥0	Mean difference between the durations of 2 consecutive GAW cycles

(C) Noise components. HI, NNE, and SPF relate the harmonic components to the noise components and are computed in the spectral space			

Harmonic Intensity (HI) [38]	%	[0; 100]	Spectrum-based ratio between the energy of harmonics components and the total energy of GAW
Harmonic to Noise Ratio (HNR) [39]	dB	(−*∞*; +*∞*)	Ratio between energies of harmonics-based signal and noise contained in GAW
Normalized Noise Error (NNE) [40]	dB	(−*∞*; +*∞*)	dB-scaled ratio between the estimated noise energy and the total energy of GAW
Spectral Flatness (SPF) [38]	dB	(−*∞*; 0]	dB-scaled difference between arithmetic and geometric means of the energy spectrum coefficients of GAW

**Table 2:** AMQ values in similar studies in comparison with a recording rate of 15 kfps in our study.

Amplitude Quotient (AMQ), healthy females							
(Ms ± SD)	Age/number of subjects	Phonation	*F*_0_	Spatial resolution (pixels)	Recording rate (fps)	Sequence length (cycles or ms)	Study
(–3.33) ± 0.73	21–45 yr/19	/i/	251 ± 31	512 × 256	4000	50 cycles	Patel et al. (2014) [41]
(–3.86) ± 1.17	22 ± 4 yr/77	Vowel	—	256 × 256	4000	250 ms	Bohr et al. (2013) [21]
(–12.09) ± 3.13	45 yr/1	/i/	176 ± 11	768 × 512	15000	106 cycles	This work

**Table 3:** Computed Closing Quotients and settings in similar studies.

Closing Quotient (CQ), healthy							
(Ms ± SD) or range	Age/number of subjects/gender	Phonation	*F*_0_	Spatial resolution (pixels)	Recording rate (fps)	Sequence length (cycles or ms)	Study
0.34	18–45 yr/18/f	/*ε*/	207 ± 16	256 × 256	4000	5 cycles	Baravieira et al. (2014) [42]
0.26 ± 0.08	20–52 yr/7/m + f	/i/	—	320 × 352	4000/6250	320–400 ms	Mehta et al. (2011) [43]
0.21–0.48	18–36 yr/20/f	/ae/	162–252	Inverse filtered airflow	8192	4 cycles	Holmberg et al. (1988) [32]

**Table 4:** GGI values in a similar study.

Glottis gap index (GGI), healthy females							
(Ms ± SD)	Age/number of subjects	Phonation	*F*_0_	Spatial resolution (pixels)	Recording rate (fps)	Sequence length (cycles)	Study
0.054 ± 0.072	28 ± 7 yr/19	/i/	251 ± 31	512 × 256	4000	50	Patel et al. (2014) [19]

**Table 5:** Open quotients and settings in similar studies.

Open quotient (OQ), healthy							
(Ms ± SD) or range	Age/number of subjects/gender	Phonation	*F*_0_	Spatial resolution (pixels)	Recording rate (fps)	Sequence length (cycles or ms)	Study
0.64 ± 0.10	20–28 yr/14/f	/i/ – healthy	—	120 × 256	2000	250 ms	Kunduk et al. (2010) [33]
0.91 ± 0.11	21–58 yr/8/m + f	/i/ – post-surgery	165 ± 58	120 × 256	2000	1000 ms	Ikuma et al. (2014) [44]
0.68–0.78	18–44 yr/26/f	/i/ – healthy	264 ± 43	160 × 140	2000	300 ms	Ahmad et al. (2012) [7]
0.79 ± 0.17	24–51 yr/16/m	/i/ – healthy	194 ± 65	256 × 256	4000	250 ms	Warhurst et al. (2014) [6]
0.44 ± 0.10	5–11 yr/#20/m + f	/i/ – healthy	287 ± 40	512 × 256	4000	30 cycles	Patel et al. (2016) [20]

**Table 6:** Shimmer values and settings in similar studies.

Shimmer SH (%), healthy							
Means	Age/number of subjects/gender	Phonation	*F*_0_	Spatial resolution (pixels)	Recording rate (fps)	Sequence length (cycles or ms)	Study
2.1	–/1/–	/i/	200	160 × 140	2000	40 cycles	Yan et al. (2005) [45]
6.2	63–82/20/f	/i/	275 ± 47	160 × 140	2000	300 ms	Ahmad et al. (2012) [24]
